# A High-Resolution Remote Sensing Image Building Extraction Model Based on Spectral Index Gated Attention and Transfer Learning

**DOI:** 10.3390/s26206492

**Published:** 2026-10-14

**Authors:** Jinchen Zhu, Shengbo Chen, Xitong Xu, Liang Cui, Yaqi Zhang, Jiaqi Yang, Qiqi Li

**Affiliations:** College of Geo-Exploration Science and Technology, Jilin University, Changchun 130026, China; zjc23@mails.jlu.edu.cn (J.Z.); xuxitong@jlu.edu.cn (X.X.); cuiliang22@mails.jlu.edu.cn (L.C.); yaqi23@mails.jlu.edu.cn (Y.Z.); jqyang23@mails.jlu.edu.cn (J.Y.); liqq23@mails.jlu.edu.cn (Q.L.)

**Keywords:** building extraction, high-resolution remote sensing imagery, spectral index gated attention, improved UNet3+, transfer learning

## Abstract

**Highlights:**

**What are the main findings?**

- We propose SIGA-UNet3+, an end-to-end building extraction framework that integrates a Spectral Index Gated Attention (SIGA) module using DSBI, NDVI, and NDWI with a source-domain pre-training and target-domain fine-tuning strategy for cross-domain building extraction.
- The multispectral index gated attention mechanism combined with transfer learning achieves the highest F1-scores (83.21% on SpaceNet and 84.56% on GF-2) and IoUs (70.19% and 72.17%) among the compared methods, demonstrating improved building extraction performance.

**What are the implications of the main findings?**

- This method provides a data-efficient solution for urban planning, land use analysis, and disaster management under limited labeled data and cross-sensor/cross-region conditions.
- The SIGA module and the transfer learning strategy provide useful insights for developing low-annotation remote sensing segmentation models under cross-domain conditions, while broader validation on additional datasets and applications remains necessary.

**Abstract:**

High-resolution remote sensing building extraction often requires large amounts of manually labeled data, and models trained on one dataset may not perform equally well on another without additional annotations. To reduce this dependency on labeled target data, we propose SIGA-UNet3+, which combines a Spectral Index Gated Attention (SIGA) module with a transfer learning strategy. The SIGA module fuses DSBI, NDVI, and NDWI to emphasize building features and suppress vegetation/water interference. Pre-training on the public SpaceNet dataset followed by fine-tuning on a self-built GF-2 dataset (Changchun, China) allows the model to adapt with limited local labels. On SpaceNet, SIGA-UNet3+ achieves F1 = 83.21% and IoU = 70.19%, obtaining the best overall segmentation performance among the compared methods. On the GF-2 dataset, it attains F1 = 84.56% and IoU = 72.17%. Moreover, using only 10% of the GF-2 training samples, transfer learning improves the F1 score by 6–8 percentage points compared to training from scratch. These results indicate that SIGA-UNet3+ can provide a promising and data-efficient approach for high-resolution remote sensing building extraction.

## 1. Introduction

With the continuous advancement of urbanization, the spatial structure and functional layout of cities have undergone significant changes [1]. As basic units of urban space, buildings and their spatial distribution, morphological characteristics, and structural information play crucial roles in urban planning, land-use management, and urban renewal [2,3]. For example, California monitors the wildland–urban interface (WUI) by detecting building footprints using high-resolution imagery and deep learning models, enabling more effective urban planning and wildfire risk management [4]. Therefore, accurate and efficient extraction of building information from high-resolution remote sensing images has become essential for smart city construction and precision urban management [5,6].

Early building extraction methods primarily relied on handcrafted features, including texture [7,8], shape [9,10], spectrum [11,12], and shadow information [13,14]. While these approaches offer relatively low computational complexity and are straightforward to implement, they are often highly sensitive to threshold selection and possess limited automation [15,16]. Consequently, they are susceptible to background interference in complex urban scenes, significantly constraining their extraction accuracy and robustness. With the widespread application of high-resolution remote sensing imagery, the increasing spectral and spatial similarity between buildings and other land covers (e.g., roads, vegetation) in high-resolution imagery further exacerbates these limitations, highlighting the need for more effective extraction approaches [17].

In recent years, the rapid development of deep learning technology has provided new solutions for automatic extraction of buildings. Convolutional Neural Networks (CNNs) can automatically learn multi-level and high-semantic feature representations through end-to-end training methods, demonstrating strong feature expression capabilities in complex background conditions [18,19]. With the proposal of Fully Convolutional Networks (FCNs), the extraction of buildings has gradually shifted from target-level detection to pixel-level semantic segmentation. Models such as UNet [20], SegNet [21], and DeepLab [22] have significantly improved extraction accuracy and spatial continuity. On this basis, researchers have further introduced multi-scale feature fusion, context modeling, and attention mechanisms, such as E-D-Net [23], AGs-UNet [24], and DSFENet [25]. By integrating local and global information or strengthening boundary and structural features, they have achieved fine-grained extraction of buildings in high-resolution remote sensing images. More recent building extraction studies have increasingly focused on transformer-based global context modeling, boundary-aware refinement, and multi-scale structural representation to improve the delineation of complex building footprints [15,23,25]. These developments indicate that current building extraction research is moving beyond conventional encoder–decoder architectures toward more specialized modeling of long-range dependencies, geometric structures, and boundary details.

Although these methods have achieved good results under controlled data conditions, their performance is highly dependent on large-scale and high-quality manual annotation data [26,27]. The significant differences in building scale, morphological characteristics, and spatial layout among different cities, as well as the inconsistencies in spatial resolution, spectral band configuration, and response characteristics of different remote sensing sensors, further weaken the robustness of the model in cross-regional and cross-sensor scenarios [28,29,30]. Especially for the high-resolution earth observation images of China’s Geographical Information System for High Resolution (GF series), due to the high cost of obtaining high-quality annotated samples and the limited data scale, directly using end-to-end deep learning models for training often fails to achieve stable and reliable extraction results, restricting the application and promotion of related methods in practical operations [31].

In conclusion, building extraction from high-resolution remote sensing images continues to face two significant challenges: (1) the heavy reliance on large-scale labeled datasets; (2) the limited adaptability across different regions and sensors [32,33,34,35]. To address these challenges, we propose SIGA-UNet3+, a novel building extraction framework that integrates a Spectral Index Gated Attention (SIGA) module with a transfer learning strategy. In cross-domain scenarios, the spectral response characteristics and spatial patterns of buildings may vary across different sensors and regions, causing conventional deep learning models to rely on domain-specific features and suffer from performance degradation [36,37]. To alleviate this problem, the SIGA module incorporates DSBI, NDVI, and NDWI as physically interpretable spectral priors to guide feature recalibration. Unlike conventional attention mechanisms that mainly learn spatial or channel-wise feature importance from training data, SIGA introduces material-related spectral information to enhance transferable building representations and reduce the influence of domain-specific background variations. Furthermore, a source-domain pre-training and target-domain fine-tuning strategy is adopted based on the UNet3+ architecture to further improve adaptation under limited labeled target-domain conditions. The key objectives of this work are: (1) to develop a method that improves building extraction accuracy under limited sample conditions, (2) to improve model adaptability under cross-domain conditions, and (3) to demonstrate the proposed method’s effectiveness through extensive experiments on both public datasets and GF-2 datasets.

## 2. Methodology

This study proposes a deep learning model for building extraction that integrates a spectral index gated attention mechanism, which is called Spectral Index Gated Attention UNet3+ (SIGA-UNet3+). SIGA-UNet3+ incorporates functionalities such as spectral feature extraction, multi-scale feature fusion, and attention guidance. It is capable of constructing physically meaningful spectral indices from input multispectral imagery and effectively fusing them with semantic features learned through deep learning. Through the adaptive modulation of feature responses by the Spectral Index Gated Attention (SIGA) module, the model can focus more intently on potential building areas during feature learning while suppressing interference from complex backgrounds, thereby enabling the accurate extraction of building targets.

### 2.1. Overall Architecture of SIGA-UNet3+

UNet3+ is selected as the backbone network because its nested and dense skip connections allow effective multi-scale feature integration and maintain spatial continuity, and its flexible encoder–decoder architecture is particularly well-suited for integrating the SIGA module, enabling the adaptive modulation of spectral index features at multiple levels of the network.

**Figure 1:**
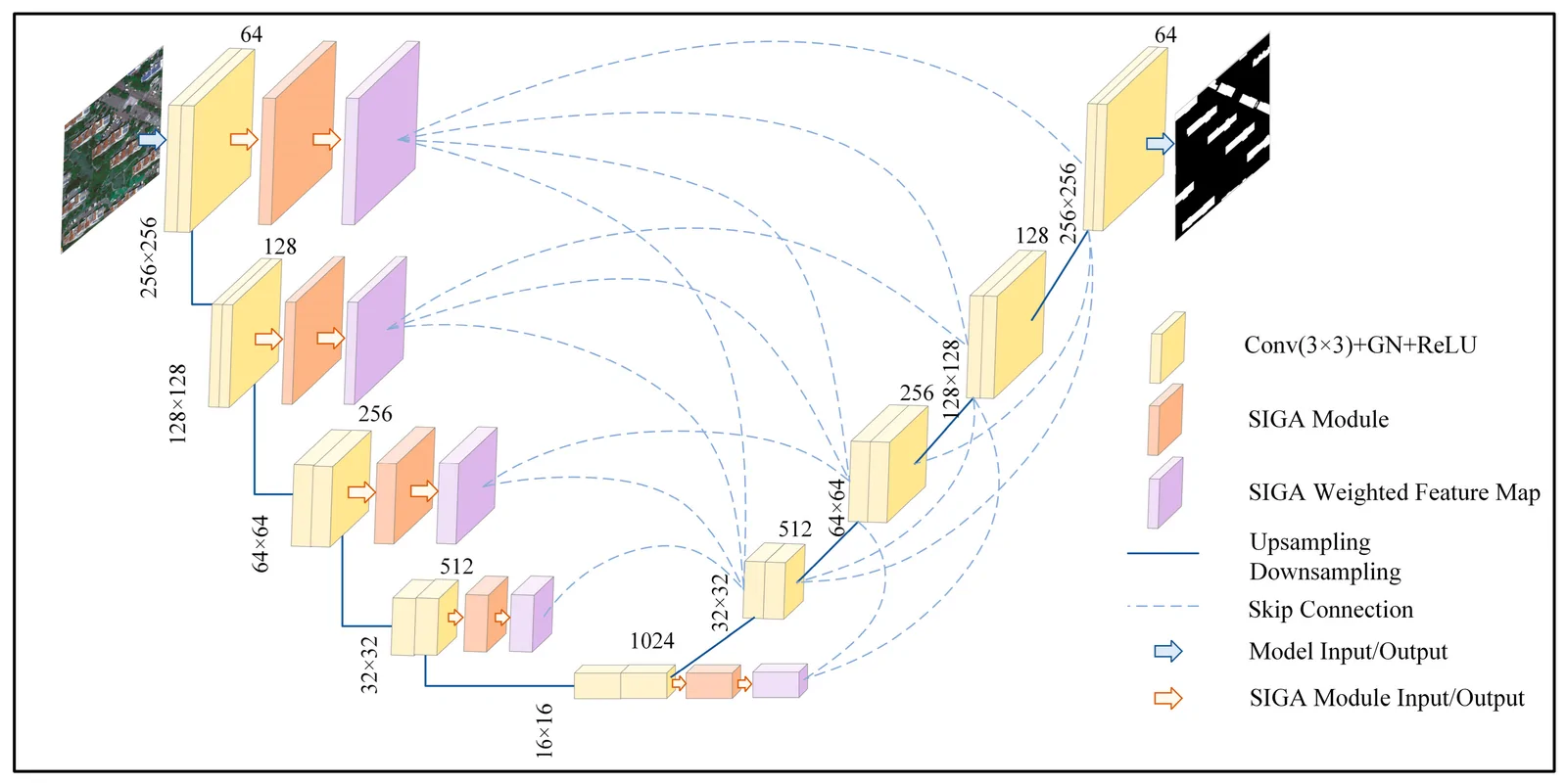
Structure of the SIGA-UNet3+ model.

The SIGA-UNet3+ model is an end-to-end building extraction model. This model retains the backbone network’s advantages in the overall structure of feature fusion across all scales. It introduces SIGA as an attention-guiding module to enhance the model’s ability to discriminate building targets and its spatial representation capabilities. As illustrated in Figure 1, the data flow proceeds as follows:

Step 1: Input and Feature Extraction:

Multispectral remote sensing images are input to the model. Multi-level semantic features are extracted through the basic convolutional modules, capturing general structures such as edges and textures that are transferable across regions and sensors. These features provide the foundation for subsequent spectral-guided attention.

**Figure 2:**
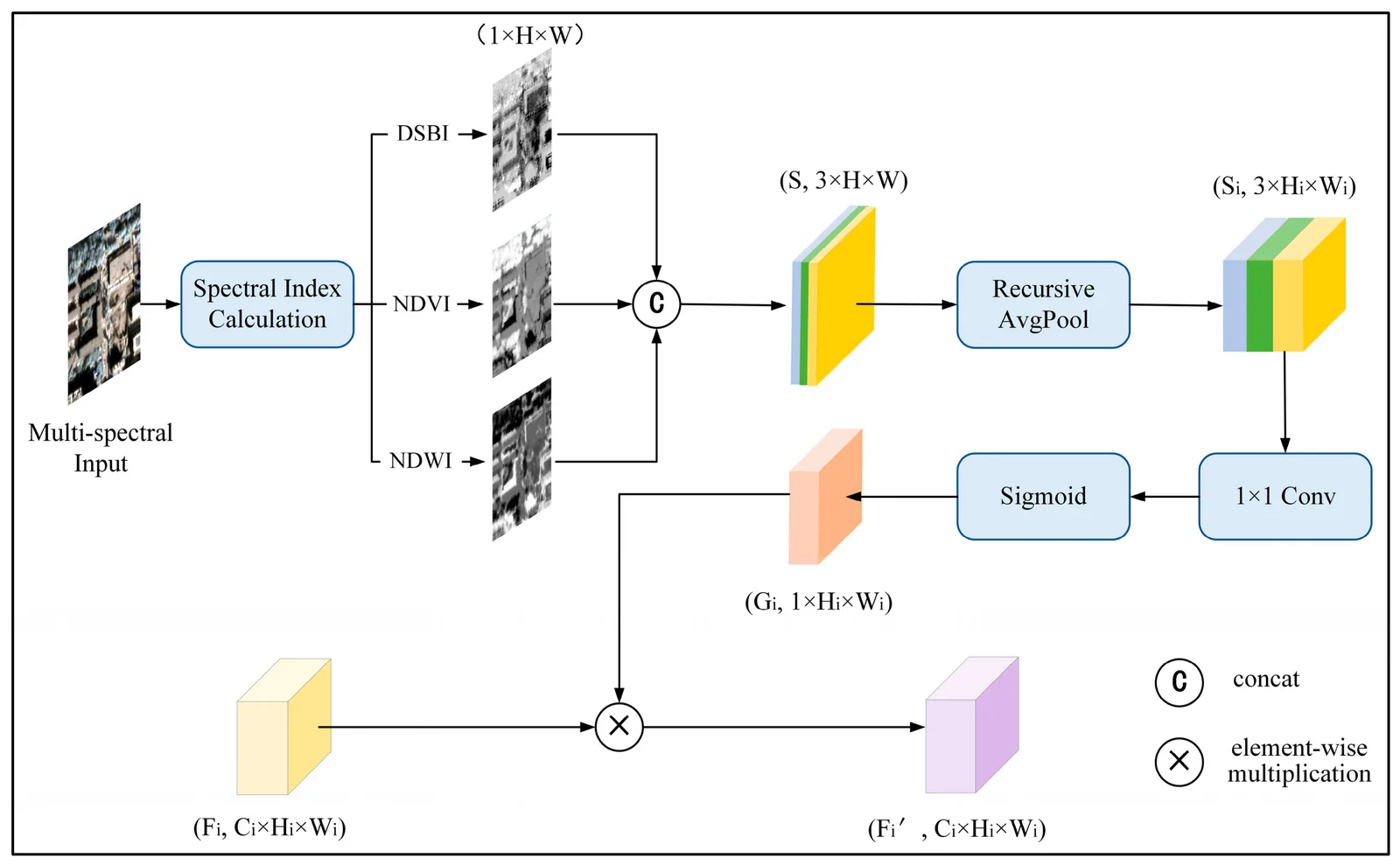
Workflow of the SIGA module.

Step 2: Spectral Index Computation (SIGA Module):

The SIGA module calculates spectral index feature maps from the input multispectral bands, including DSBI, NDVI, and NDWI. These indices have clear physical meanings and enhance building-specific signals while suppressing background features such as vegetation and water (Figure 2). The spectral indices are then mapped to match the spatial scales of the intermediate deep features.

Step 3: Feature Fusion and Attention Guidance:

The spectral index features are fused with deep features from intermediate layers of UNet3+, and a gating mechanism adaptively modulates channel responses. This allows the network to focus on potential building areas and suppress background noise, which is particularly important under small-sample conditions. By explicitly introducing spectral prior knowledge, SIGA reduces feature confusion caused by similar spectral responses in non-building areas.

Step 4: Decoding and Multi-scale Feature Integration:

In the decoding stage, full-scale skip connections of UNet3+ integrate features across all levels, restoring spatial structure and boundary information of buildings [36,38]. This multi-scale fusion ensures spatial continuity and enhances the model’s adaptability across regions and sensors.

Step 5: Output:

The output layer generates pixel-level building predictions. All modules are supervised through pixel-wise annotations, supporting accurate and stable extraction even in complex urban environments. Each component is explicitly designed to mitigate small-sample limitations and improve cross-domain adaptability, as highlighted in Figure 1.

### 2.2. Architecture of SIGA Module

Deep learning models typically rely on data-driven semantic features, which can lead to confusion when buildings share similar spectral characteristics with roads, bare land, or water, especially under cross-region or cross-sensor conditions. This problem is exacerbated when training samples are limited.

To address the issues above, this study introduces spectral indices as prior information with explicit physical meaning and designs a Spectral Index Gated Attention (SIGA) module to guide the deep learning network to focus more on potential building areas during feature learning. SIGA module combines spectral index information with the features of the network’s intermediate layers, thereby maintaining the advantages of the original network structure while introducing spectral constraints that are discriminative for buildings. The workflow of the SIGA module is as illustrated in Figure 2.

Step 1: Spectral Index Construction:

The SIGA module first constructs spectral indices from the input multispectral image to enhance discriminative features for buildings and suppress background interference. The choice of these three indices is motivated by their complementary physical meanings and proven effectiveness in remote sensing. The three indices were selected to provide complementary spectral evidence from two directions: DSBI serves as a positive prior for enhancing built-up features, whereas NDVI and NDWI serve as exclusionary priors for identifying two major sources of background confusion, namely vegetation and water. Therefore, their combination provides both target-enhancement and background-suppression information rather than relying on a single spectral cue.

Difference-based Spectral Built-up Index (DSBI) [11] was originally designed for high-resolution imagery lacking SWIR bands. Buildings generally exhibit distinct reflectance characteristics in visible wavelengths compared with vegetation, while vegetation usually shows lower red reflectance due to chlorophyll absorption. Therefore, the spectral contrasts between blue/green and red bands can provide useful information for distinguishing impervious surfaces from vegetated backgrounds. Based on this observation, DSBI integrates differential information from blue–red and blue–green bands to enhance the spectral contrast of built-up areas. Compared with using a single band difference, the combination of multiple visible-band differences provides complementary spectral cues and reduces the dependence on a specific band relationship, which can facilitate more reliable building feature representation.

In addition to DSBI, this study also incorporates the Normalized Difference Vegetation Index (NDVI) [39] and the Normalized Difference Water Index (NDWI) [40] to suppress non-building areas, such as vegetation and water bodies. The NDVI is widely used for vegetation detection, as it effectively distinguishes vegetated areas from non-vegetated areas by highlighting differences in reflectance between the red and near-infrared bands. Similarly, the NDWI is effective for delineating water bodies, as it exploits the differences in reflectance between the green and near-infrared bands to enhance water features while minimizing the influence of other surface types.

The combination of DSBI, NDVI, and NDWI provides complementary spectral cues for distinguishing buildings from major background classes in high-resolution imagery. Specifically, DSBI acts primarily as a building-enhancement prior by emphasizing the visible-band spectral contrast of built-up surfaces, whereas NDVI and NDWI provide background-suppression cues by identifying vegetation- and water-related responses, respectively. Their roles are therefore not redundant: DSBI increases the sensitivity to potential building regions, while NDVI and NDWI reduce false responses from spectrally confusing non-building areas. By combining these positive and exclusionary priors, the SIGA module can generate more discriminative spectral guidance for subsequent feature recalibration. These three indices are normalized to the range [0,1] using image-level Min-Max normalization and concatenated along the channel dimension to form a multi-index feature tensor *S*. This normalization is applied after spectral index calculation and aims to align the numerical ranges of different spectral priors (DSBI, NDVI, and NDWI) before fusion in the SIGA module. Since the spectral indices have different value distributions, this operation enables the attention module to effectively utilize their relative spatial contrast information rather than being dominated by a specific index due to scale differences. This explicit use of spectral priors provides informative spectral cues for distinguishing buildings, especially important when training data are limited. The calculation formulas for the three spectral indices (Equations (1)–(3)) and Min-Max normalization (Equations (4) and (5)) are as follows:

*DSBI* = 0.5 × (*B* − *R*) + 0.5 × (*B* − *G*), (1)

(2) $$\mathrm{NDVI}=\frac{\mathrm{NIR}-R}{\mathrm{NIR}+R},$$

(3) $$\mathrm{NDWI}=\frac{G-\mathrm{NIR}}{G+\mathrm{NIR}},$$

where *B*, *G*, *R*, and *NIR* represent the reflectance values of the blue, green, red, and near-infrared spectral bands, respectively.

(4) $$\hat{SI_{k}}(i,j)=\frac{SI_{k}(i,j)-\min(SI_{k})}{\max(SI_{k})-\min(SI_{k})},$$

where *SI_k_*(*i*,*j*) is the original value of the *k*-th spectral index *SI* at spatial position (*i*,*j*), and *min*(*SI_k_*) and max(*SI_k_*) denote the minimum and maximum values of the *k*-th spectral index within the current image.

The normalized spectral indexes are then concatenated along the channel dimension to form a multi-index feature tensor *S*:

*S* = *Concat*(*DSBI*, *NDVI*, *NDWI*) ∈ *R*^3×*H*×*W*^, (5)

Step 2: Scale Alignment:

To ensure spatial alignment between the multi-index feature tensor and the intermediate features of UNet3+, the SIGA module employs recursive average pooling for scale mapping of the multi-index feature tensor. By applying a 2 × 2 average pooling operation to the spectral features of the previous scale, feature tensors with decreasing resolution are generated layer by layer. The feature tensor of the *i*-th layer *S_i_* can be expressed as:

(6) $$S_{i}=\left\{\begin{array}{l} S\;\;\;\;\;\;\;i=1\\ \mathrm{AvgPoo}l_{2\times 2}(S_{i-1})\;i=2,3,4,5 \end{array}\right.,$$

Step 3: Attention Gating and Feature Fusion:

Since the gating weights are primarily intended to represent the spatial attention distribution, a single-channel representation is sufficient to effectively reflect the feature intensity in building areas, while also avoiding the additional computational overhead and training instability associated with multi-channel gating. Therefore, the final spectral gated weights of the *i*-th layer are generated through 1 × 1 convolution and the Sigmoid activation function as *G_i_*:

(7) $$G_{i}=\sigma (f_{\mathrm{conv}}(S_{i}))=\frac{1}{1+e^{-f_{\mathrm{conv}}(S_{i})}}\in R^{1\times H_{i}\times W_{i}},$$

where *f_conv_* refers to the 1 × 1 convolutional operation applied to the feature map *S_i_* in the *i*-th layer. This convolution operation serves to reduce the dimensionality of the input features by combining them into a single-channel representation, which is then passed through the Sigmoid activation function *σ* to generate the gating weights. The use of 1 × 1 convolution ensures that only the most important spatial features are retained while minimizing computational complexity. Assuming the feature map of the *i*-th layer in the UNet3+ network is denoted as *F_i_*, the computation process of the SIGA module can be expressed as:

(8) $$F_{i}^{\prime }=F_{i}⊗G_{i},\;F_{i}^{\prime }\in R^{C_{i}\times H_{i}\times W_{i}},$$

where ⊗ denotes element-wise multiplication, and *G_i_* is spatially aligned with *F_i_*.

Unlike conventional channel- or spatial-attention modules, which derive attention weights primarily from learned feature representations, SIGA introduces physically interpretable spectral priors derived from DSBI, NDVI, and NDWI. These spectral priors are spatially aligned with the intermediate feature maps and transformed into learnable gating weights, allowing the network to recalibrate features using both data-driven semantic information and material-related spectral cues. Therefore, the main distinction of SIGA lies in the source of attention guidance: conventional attention is primarily feature-driven, whereas SIGA explicitly incorporates physically meaningful spectral information into the attention generation process. This design may help reduce the dependence on domain-specific appearance patterns and facilitate adaptation across sensors and regions.

### 2.3. Loss Function Design

To address the imbalance in pixel distribution between the foreground and background in the building extraction task, this study employs a weighted combination of Binary Cross-Entropy (BCE) Loss and Dice Loss as the optimization objective during model training. The BCE Loss is used to constrain pixel-level classification accuracy, while the Dice Loss focuses on enhancing the overall overlap between the predictions and the ground truth building areas. The two loss functions complement each other, contributing to improved model stability and segmentation performance.

The BCE loss and Dice loss are defined as follows:

(9) $$L_{\mathrm{BCE}}=-\frac{1}{N}\sum_{i=1}^{N}\left[y_{i}\log(p_{i})+(1-y_{i})\log(1-p_{i})\right],$$

(10) $$L_{\mathrm{Dice}}=1-\frac{2\sum_{i=1}^{N}p_{i}y_{i}+\varepsilon }{\sum_{i=1}^{N}p_{i}+\sum_{i=1}^{N}y_{i}+\varepsilon },$$

where *y_i_* = {0, 1} denotes the ground truth label of the *i*-th pixel, *p_i_* = {0, 1} represents the probability predicted by the model that the pixel belongs to a building, and *N* is the total number of pixels.

By assigning equal weights to BCE and Dice losses, the optimization objective gives comparable importance to pixel-level classification and region-level overlap. In this study, the weights were empirically set to 0.5 and 0.5 following commonly used balanced combinations in semantic segmentation tasks [25,41,42]. This setting provides a simple and stable baseline for jointly optimizing local classification accuracy and global region consistency. However, the equal-weight configuration was not selected through an exhaustive weight-sensitivity search and should therefore not be interpreted as the theoretically optimal combination. Therefore, the overall loss function for SIGA-UNet3+ is expressed as:

*L* = 0.5*L_BCE_* + 0.5*L_Dice_*, (11)

### 2.4. Evaluation Metrics

To comprehensively evaluate the model’s performance in the building extraction task, this study adopts Precision, Recall, F1-score, Intersection over Union (IoU), Overall Accuracy (OA) and Kappa as evaluation metrics. All of the above metrics are calculated based on pixel-level classification results, with the building category designated as the positive sample and the non-building category as the negative sample.

Precision reflects the accuracy of the model in identifying building areas; Recall measures the model’s ability to fully extract building targets; F1 comprehensively considers both Precision and Recall, whereas IoU more intuitively reflects the degree of spatial overlap between the predicted results and the ground truth. In addition, OA measures the proportion of correctly classified pixels among all pixels, providing a straightforward indicator of overall performance, and the Kappa coefficient quantifies the agreement between predicted results and ground truth while accounting for chance agreement, offering a more robust assessment of classification reliability. The evaluation metrics are defined as follows:

(12) $$\mathrm{Precision}=\frac{\mathrm{TP}}{\mathrm{TP}+\mathrm{FP}},$$

(13) $$\mathrm{Recall}=\frac{\mathrm{TP}}{\mathrm{TP}+\mathrm{FN}},$$

(14) $$F1=\frac{2\times \mathrm{Precison}\times \mathrm{Recall}}{\mathrm{Precison}+\mathrm{Recall}},$$

(15) $$\mathrm{IoU}=\frac{\mathrm{TP}}{\mathrm{TP}+\mathrm{FP}+\mathrm{FN}},$$

(16) $$\mathrm{OA}=\frac{\mathrm{TP}+\mathrm{TN}}{\mathrm{TP}+\mathrm{TN}+\mathrm{FP}+\mathrm{FN}},$$

(17) $$\kappa =\frac{P_{o}-P_{e}}{1-P_{e}},$$

(18) $$P_{e}=\frac{\left(\mathrm{TP}+\mathrm{FP})(\mathrm{TP}+\mathrm{FN})+(\mathrm{FN}+\mathrm{TN})(\mathrm{FP}+\mathrm{TN}\right)}{{\left(\mathrm{TP}+\mathrm{FN}+\mathrm{FP}+\mathrm{FN}\right)}^{2}},$$

where TP denotes the number of pixels correctly identified as buildings, TN denotes the number of pixels correctly identified as background, FP denotes the number of pixels misclassified as buildings, and FN denotes the number of missed building pixels. TP, TN, FP, and FN are accumulated from all pixels in the entire test set, and all evaluation metrics are calculated based on this aggregated pixel-level confusion matrix.

In addition to the conventional region-based metrics, Boundary IoU (BIoU) was introduced to quantitatively evaluate building boundary accuracy and geometric shape preservation. For a binary building mask *M*, *B_d_*(*M*) denotes the boundary region within a predefined distance d from its contour. Given the predicted building mask *P* and the corresponding ground-truth mask *G*, BIoU is calculated as:

(19) $$\mathrm{BIoU}=\frac{\left|B_{d}(P)\cap B_{d}(G)\right|}{\left|B_{d}(P)\cup B_{d}(G)\right|},$$

where *B_d_*(*P*) and *B_d_*(*G*) denote the boundary regions of the predicted and ground-truth masks, respectively. In this study, *d* was set to 2 pixels. Unlike conventional IoU, which measures the overlap of the entire building regions, BIoU focuses specifically on boundary agreement and is therefore more sensitive to contour displacement and local geometric distortion. A higher BIoU indicates better consistency between the predicted and reference building boundaries.

### 2.5. Experimental Setup

To evaluate the effectiveness and cross-domain adaptability of SIGA-UNet3+, experiments were designed under both small-sample and domain-shift conditions. Specifically, the model was pre-trained on the SpaceNet dataset and subsequently transferred to the GF-2 dataset, introducing variations in sensor characteristics, spectral responses, spatial resolution, and geographic environment. The pre-training stage employs the SpaceNet Shanghai building dataset [43]. It consists of WV-3 multispectral remote sensing images and corresponding vector annotations of buildings. This dataset has a wide area coverage and features a diverse range of building types, providing the model with rich and representative building feature information.

A self-built GF-2 buildings dataset of the Changchun area is employed in the transfer learning stage. This dataset is constructed based on the high-resolution multispectral images of Chaoyang District, Changchun City, Jilin Province. The images are obtained by the GF-2 series satellites, and the building annotations are mainly produced through visual interpretation. The GF-2 image has a high spatial resolution, and the target area features a complex urban structure. It is an ideal research area for verifying the building extraction capability of the spectral index attention module and the transfer learning strategy in complex urban areas. Therefore, the transfer from SpaceNet to GF-2 provides a realistic cross-domain evaluation scenario for assessing the adaptability of the proposed model under different sensor and regional conditions.

#### 2.5.1. Data Processing

For the SpaceNet dataset, since the WV-3 images employed contain 8 multispectral bands while the GF-2 images only have 4 bands, in order to ensure the consistency of the input features during the transfer learning process, the bands of the SpaceNet images are reorganized according to the band configuration of GF-2, and only the blue, green, red and near-infrared bands are retained as the input for the model. Meanwhile, in order to reduce the influence of the difference in feature scales under different spatial resolutions on the model’s transfer effect, the SpaceNet images (with a native spatial resolution of 0.31 m) are uniformly resampled to a spatial resolution of 0.8 m, which is the same as that of GF-2 imagery. For the GF-2 multispectral images, radiation calibration, orthorectification, and fusion processing of panchromatic and multispectral images were carried out successively. Under the premise of maintaining stable spectral characteristics, the spatial resolution was improved to 0.8 m, thereby enhancing the expression ability of building boundaries and detailed structures.

After completing the geometric and radiative preprocessing, fine annotations of buildings are obtained through visual interpretation. Then the vector annotations are rasterized to generate building mask data that are consistent with the spatial resolution and coordinate system of the training images. To ensure consistent model input and prevent data leakage, the original images were first spatially partitioned into independent training, validation, and test regions with a ratio of 70%, 15%, and 15%, respectively. Subsequently, each region was cropped into 256 × 256 patches for model training and evaluation [44]. A fixed patch size was used throughout all experiments to ensure consistent input dimensions and fair comparisons among different models and training strategies.

A data augmentation strategy is also introduced during the model training stage to enhance the stability of the training process. This strategy includes random rotations within the range of [−30°, 30°], horizontal and vertical flipping with a probability of 0.5, and random scaling within the range of 0.8 to 1.2, followed by random cropping to maintain a fixed input size. These transformations increase the diversity of the training samples without altering the semantic and spatial structure of the buildings. Data augmentation was performed using geometric transformations, including rotation, flipping, scaling, and cropping. The number of augmented samples was increased by approximately 50% relative to the original training set, providing additional spatial variations while preserving the original data distribution. These geometric transformations help reduce overfitting and improve the model’s adaptability to unseen scenes.

All input images were normalized by subtracting the mean and dividing by the standard deviation computed from the training dataset for each channel. The normalization reduces radiometric differences and stabilizes network optimization and is performed as follows:

(20) $$x_{\mathrm{norm}}=\frac{x-\mu }{\sigma },$$

where *x* represents the pixel values of the image, and *μ* and *σ* are the mean and standard deviation for each channel.

#### 2.5.2. Training Procedure

The model training and testing are carried out under the Windows 11 operating system using PyTorch 2.0.1. The experimental platform is equipped with an Intel^®^ Xeon^®^ Gold 5218 CPU (Intel Corporation, Santa Clara, CA, USA), 256 GB of memory, and a 24 GB NVIDIA RTX 3090 GPU (NVIDIA Corporation, Santa Clara, CA, USA). During the model training process, the AdamW optimizer is employed with an initial learning rate of 10^−4^, β_1_ = 0.9, β_2_ = 0.999 and a weight decay of 0.01, and the cosine annealing learning rate decay strategy is adopted. The batch size was set to 32, the pre-training period was 100 epochs, and the transfer learning training period was 20 epochs. Model weights were initialized using Kaiming normal initialization to ensure stable convergence.

During transfer learning, the pre-trained SIGA-UNet3+ model was adapted to the GF-2 dataset. The encoder of UNet3+ was frozen to preserve general low-level and mid-level visual representations learned from the source domain, while the decoder and SIGA module remained trainable. Since SIGA operates on the intermediate feature maps extracted by the frozen encoder, the attention parameters can still be optimized to learn target-domain-specific feature weighting without modifying the original encoder representations. Therefore, the fine-tuning process updates the parameters of the SIGA module and decoder through gradient backpropagation, allowing the spectral gating weights to be optimized according to target-domain characteristics.

To ensure the reproducibility and reliability of the experiments, fixed random seeds were used across all operations. Each configuration was repeated three times with these different random seeds to minimize the impact of stochastic variations. The results were then averaged to ensure consistency and eliminate biases due to different random initializations. This approach ensures robust performance evaluation and mitigates the influence of random fluctuations.

Early stopping was applied during training to prevent overfitting. Training was halted if the validation loss did not improve for 5 consecutive epochs, and the model weights from the epoch with the lowest validation loss were restored.

Model checkpoints were saved every 10 epochs to ensure that training could be resumed from the most recent checkpoint in case of any interruptions or issues.

## 3. Results

### 3.1. Pre-Training Performance

#### 3.1.1. Comparative Results on the SpaceNet Dataset

To provide a representative comparison across different segmentation paradigms, we selected UNet3+, SegNet [21], DeepLabv3+ [22,45], and Swin Transformer [46,47] as the baseline models. UNet3+ and SegNet represent conventional encoder–decoder CNN architectures, DeepLabv3+ represents multi-scale context modeling based on dilated convolution and ASPP, and Swin Transformer provides a recent transformer-based reference with enhanced long-range dependency modeling. Together, these models cover several mainstream architectural paradigms and provide a consistent basis for evaluating the contribution of the proposed spectral-guided attention mechanism.

Table 1 reports the quantitative results on the SpaceNet dataset. SIGA-UNet3+ achieves the best overall performance, with F1 = 83.21%, IoU = 70.19%, OA = 85.46%, and Kappa = 71.36%. Compared with the baseline UNet3+, SIGA-UNet3+ improves F1, IoU, OA, and Kappa by 5.54, 5.04, 4.34, and 10.15 percentage points (pp), respectively. Compared with the second-best model (Swin Transformer), SIGA-UNet3+ achieves improvements of 1.24 pp in F1, 1.74 pp in IoU, 1.44 pp in OA, and 3.01 pp in Kappa. Although Swin Transformer achieves slightly higher Precision than SIGA-UNet3+ (85.12% vs. 84.73%), SIGA-UNet3+ obtains substantially higher Recall (81.74% vs. 78.96%). This reflects a Precision–Recall trade-off: Swin Transformer produces relatively conservative predictions with fewer false positives, whereas SIGA-UNet3+ recovers a larger proportion of true building pixels. Consequently, SIGA-UNet3+ achieves higher F1-score and IoU, indicating a better overall balance between omission and commission errors. The additional statistical analysis in Appendix A (Table A1) further demonstrates the stability of these improvements, with SIGA-UNet3+ achieving the highest F1-score (83.21 ± 1.0%) and IoU (70.19 ± 1.8%) across repeated experiments. Moreover, the effect sizes between SIGA-UNet3+ and Swin Transformer are 1.12 for F1-score and 0.84 for IoU, indicating practically meaningful improvements over the strongest baseline included in this study.

**Figure 3:**
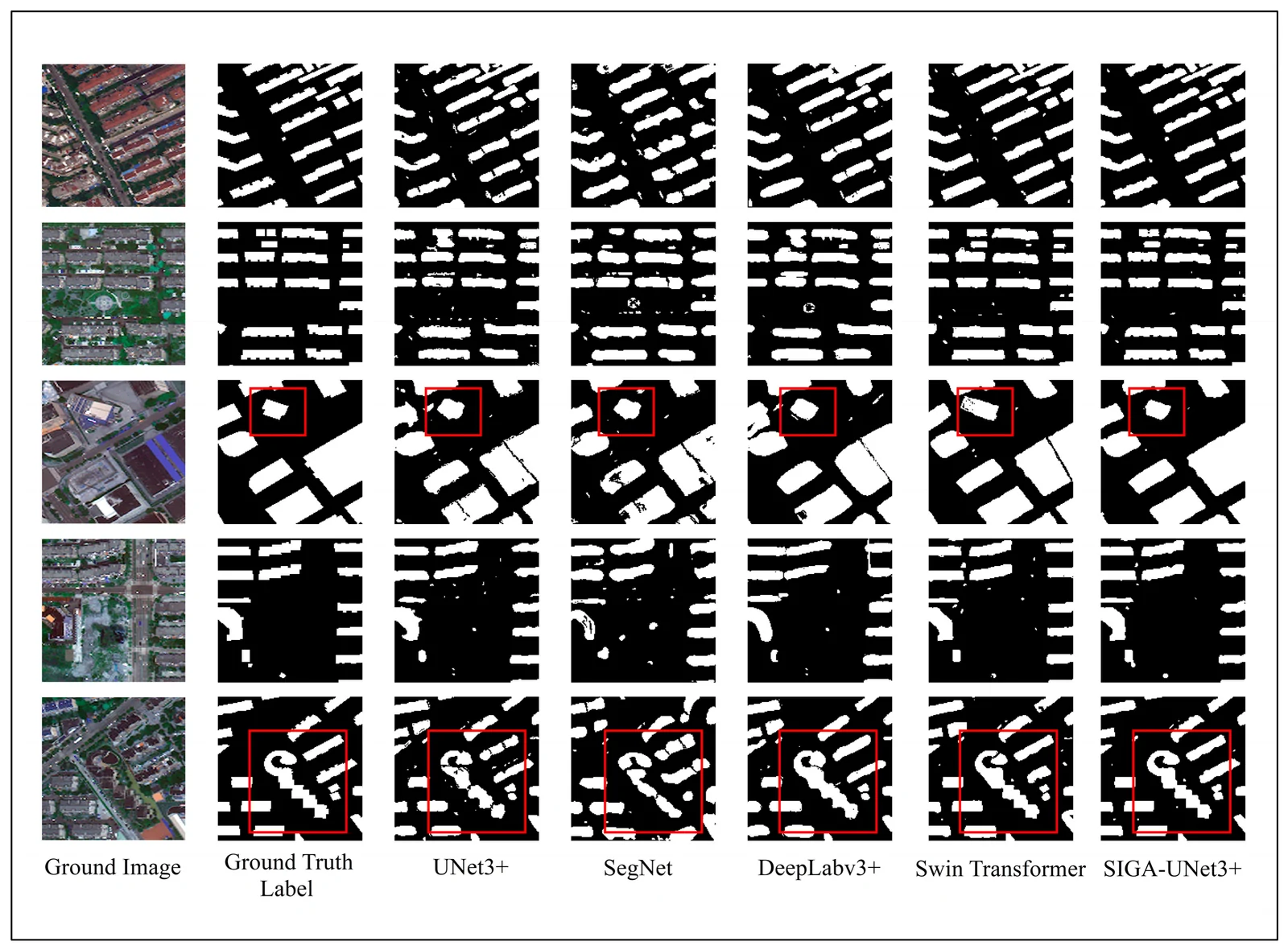
Predictions of different models on SpaceNet dataset. Red rectangles highlight regions where the SIGA-UNet3+ produces more continuous boundaries and finer spatial details compared to other models.

Visual results in Figure 3 further support the quantitative comparison. SIGA-UNet3+ generally produces more complete building regions and better-defined contours than the conventional CNN-based baselines, while Swin Transformer also demonstrates competitive boundary delineation. These results provide qualitative evidence of the proposed model’s segmentation capability on the source-domain dataset.

#### 3.1.2. Ablation Studies

**Figure 4:**
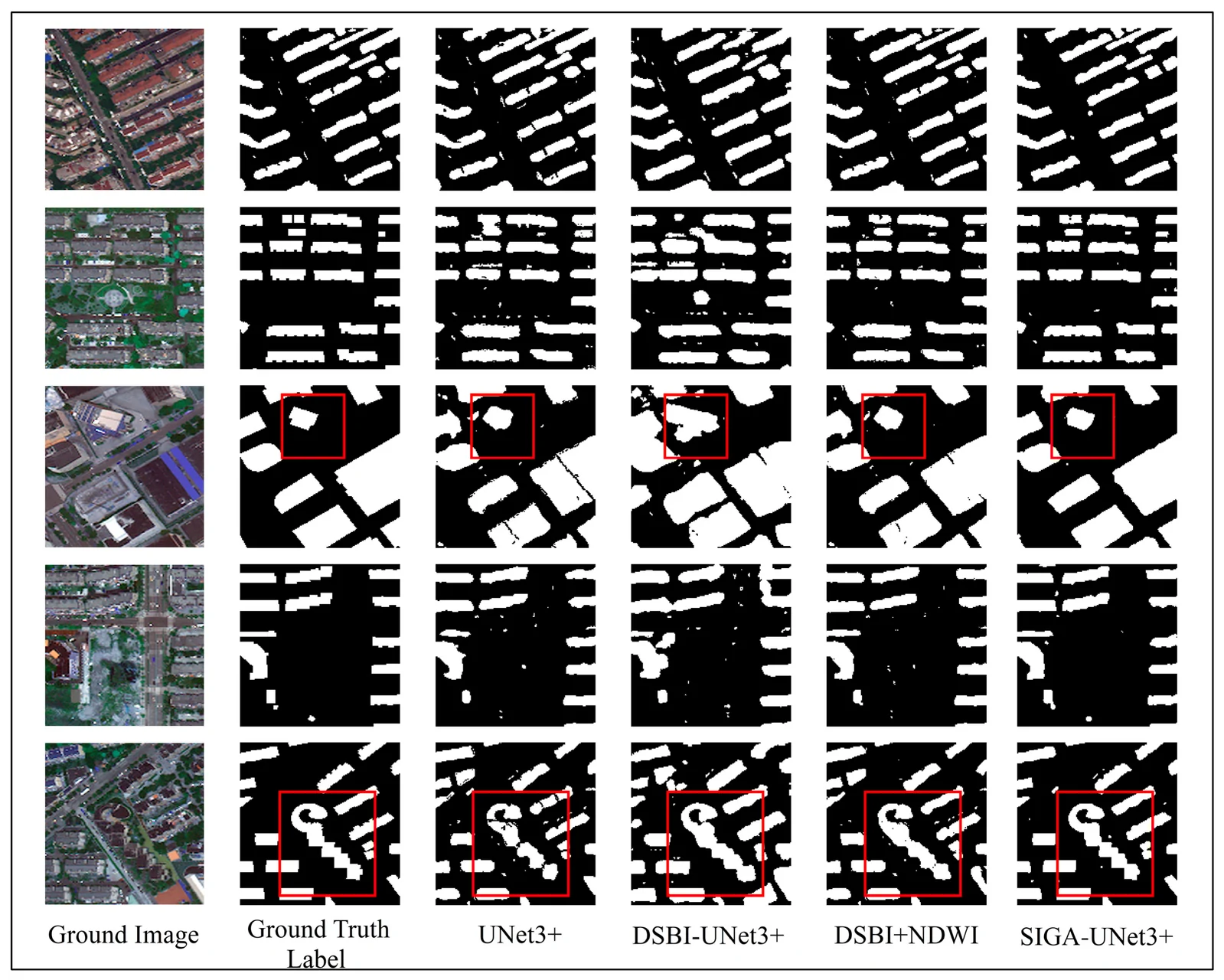
Predictions of ablation models. Red rectangles indicate representative regions with complex building structures and local boundary details used for boundary and shape analysis.

To evaluate the contribution of each spectral index and their combinations, we performed ablation experiments on the SpaceNet dataset using UNet3+ as the baseline. Table 2 includes single-index models (DSBI, NDVI, NDWI), two-index combinations (DSBI + NDVI, DSBI + NDWI, NDVI + NDWI), and the full SIGA-UNet3+ integration. Table 2 and Figure 4 demonstrate the unique roles of different spectral indices in the SIGA module as well as their overall collaborative effects.

Table 2 reports the ablation results on the SpaceNet dataset. Compared to the baseline UNet3+, every variant with a single spectral index (DSBI, NDVI, or NDWI) yields higher F1 and IoU. Specifically, DSBI-UNet3+ achieves F1 = 81.24% and IoU = 68.86%, representing improvements of 3.57 pp in F1 and 3.71 pp in IoU over UNet3+. NDVI-UNet3+ improves F1 by 2.89 pp, and NDWI-UNet3+ improves F1 by 1.87 pp.

Among single-index variants, DSBI-UNet3+ attains the highest Recall (83.31%) and IoU (68.86%), while NDVI-UNet3+ achieves the highest Precision (82.54%). The integration of three indices (SIGA-UNet3+) further improves performance, reaching F1 = 83.21% and IoU = 70.19%. Compared to the best single-index model (DSBI-UNet3+), SIGA-UNet3+ improves F1 by 1.97 pp and IoU by 1.33 pp; relative to UNet3+, the gains are 5.54 pp in F1 and 5.04 pp in IoU. In terms of OA and Kappa, SIGA-UNet3+ achieves gains of 4.34 pp in OA and 10.15 pp in Kappa over UNet3+.

Visual results in Figure 4 (red rectangles) support these quantitative comparisons. In regions where single-index models show incomplete building areas or residual background noise, SIGA-UNet3+ produces more continuous boundaries and fewer false positives, consistent with its higher Recall and IoU.

#### 3.1.3. Computational Cost Analysis

Table 3 reports the model complexity and inference efficiency of all evaluated methods (image size 256 × 256). SIGA-UNet3+ contains 27.5 M parameters, which is 0.4 M (1.5%) more than the baseline UNet3+ (27.1 M), 1.6 M (5.5%) fewer than Swin Transformer (28.3 M), 2.0 M (6.8%) fewer than SegNet (29.5 M), and 0.3 M (1.1%) fewer than DeepLabv3+ (27.8 M). In terms of computational complexity, SIGA-UNet3+ requires 79.1 G FLOPs, which is comparable to UNet3+ (78.3 G) and 14.6% lower than Swin Transformer (92.7 G). The inference time of SIGA-UNet3+ is 27.3 ms/image, which is 1.5× faster than Swin Transformer (41.5 ms) and 1.4× faster than SegNet (38.2 ms), while being only 0.5 ms slower than UNet3+ (26.8 ms).

These results indicate that the proposed SIGA-UNet3+ achieves competitive segmentation performance with minimal additional computational overhead relative to the baseline UNet3+, while remaining more efficient than the transformer-based and heavier CNN-based models. Since both the SpaceNet and GF-2 datasets are processed using the same 256 × 256 input size and identical model configurations, the number of parameters and FLOPs are unchanged across the two datasets, and the per-patch inference cost is expected to remain comparable under the same hardware environment. Therefore, the computational-cost results in Table 3 are also representative of the GF-2 experiments.

### 3.2. Transfer Learning Performance

#### 3.2.1. Comparative Results on the GF2 Dataset

**Figure 5:**
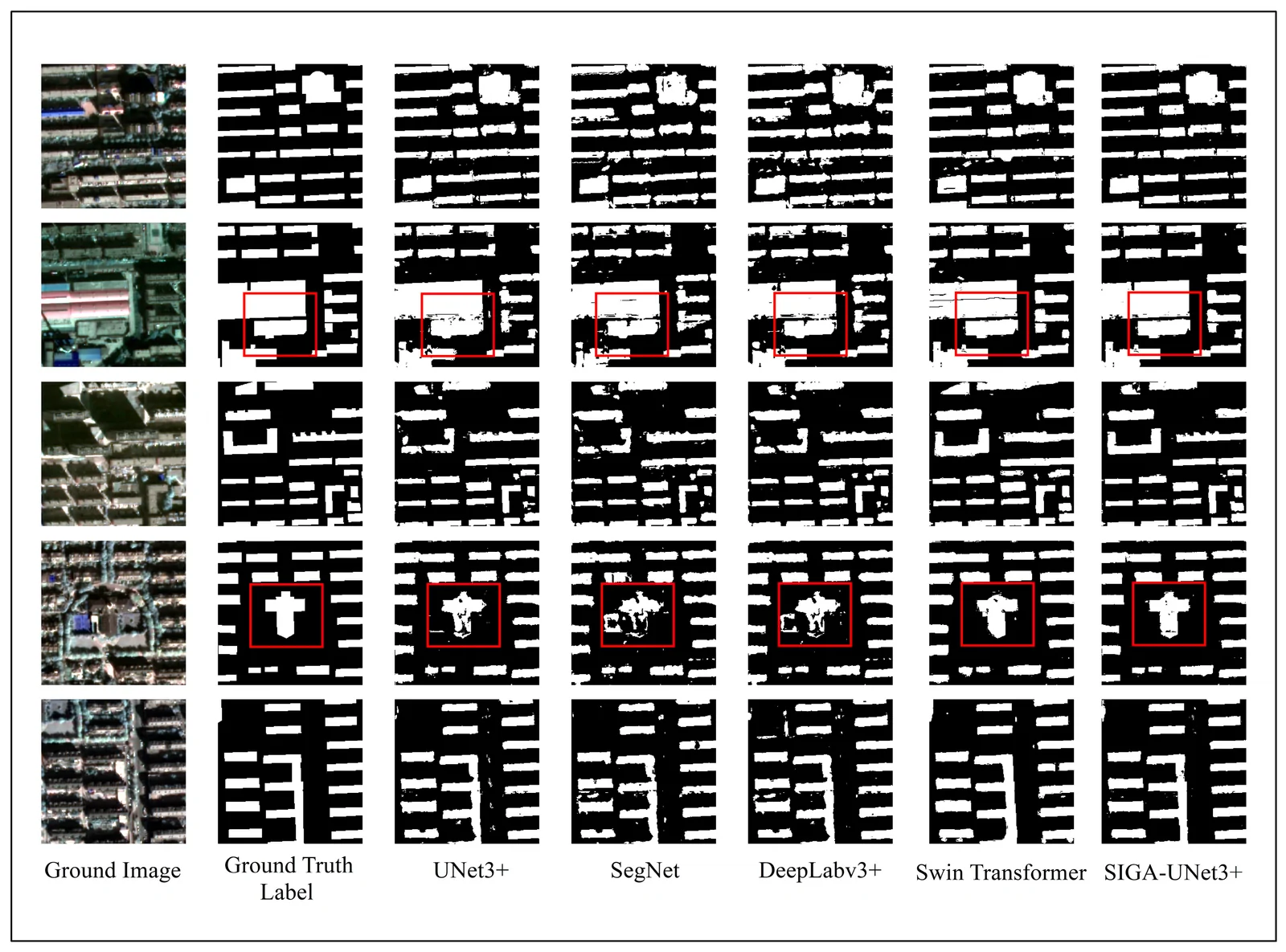
Predictions of different models on GF-2 dataset. Red rectangles indicate representative regions with complex building structures and local boundary details used for boundary and shape analysis.

On the GF-2 Changchun dataset, the building extraction performance of different models was further compared after transfer learning. Table 4 presents the conventional region-based evaluation results, Table 5 reports the boundary-oriented evaluation using BIoU, and Figure 5 shows representative prediction results of the compared models.

Table 4 reports the quantitative results on the GF-2 Changchun dataset after transfer learning. SIGA-UNet3+ achieves F1 = 84.56%, IoU = 72.17%, OA = 86.63%, and Kappa = 73.06%. Compared to the baseline UNet3+, SIGA-UNet3+ improves F1 by 5.74 pp, IoU by 5.45 pp, OA by 4.46 pp, and Kappa by 9.42 pp. Compared with the second-best model, Swin Transformer, SIGA-UNet3+ yields gains of 1.03 pp in F1, 2.05 pp in IoU, 1.47 pp in OA, and 3.23 pp in Kappa. Although its Precision is slightly lower than that of Swin Transformer (86.15% vs. 87.45%), SIGA-UNet3+ achieves higher Recall (83.02% vs. 80.23%), indicating a stronger ability to recover complete building regions on the target GF-2 dataset. This advantage is reflected in its higher F1-score and IoU. The statistical analysis in Appendix A (Table A2) further confirms the consistent performance of SIGA-UNet3+ after domain adaptation. The model maintains relatively low variations across repeated experiments, achieving the highest F1-score (84.56 ± 1.1%) and IoU (72.17 ± 1.9%) among all compared methods. Compared with Swin Transformer, SIGA-UNet3+ obtains large effect sizes for both F1-score (Cohen’s d = 0.86) and IoU (Cohen’s d = 0.98), suggesting that the observed improvements remain practically meaningful on the target GF-2 dataset.

The boundary-oriented results in Table 5 further support the advantage of SIGA-UNet3+. It achieves the highest BIoU of 64.79%, exceeding UNet3+ and Swin Transformer by 9.50 and 3.52 percentage points, respectively. This indicates that the improvement of SIGA-UNet3+ is not limited to region-level overlap but also extends to boundary consistency. The qualitative results in Figure 5 are consistent with this finding: compared with the other models, SIGA-UNet3+ better preserves local geometric details, including corners, protrusions, recesses, and abrupt directional changes, particularly for zigzag- and sawtooth-shaped buildings that tend to be smoothed by conventional CNN-based methods. In addition, SIGA-UNet3+ produces more continuous contours and more complete building regions, which is consistent with the higher Recall and IoU reported in Table 4.

It should be noted that the slightly higher quantitative performance on GF-2 does not imply that the GF-2 scenes are intrinsically easier than those in SpaceNet. The SpaceNet results correspond to the source-domain pre-training stage, whereas the GF-2 results are obtained after source-domain pre-training and target-specific fine-tuning. Moreover, cross-dataset differences in pixel-level metrics can also be affected by building size and density, foreground–background distribution, scene composition, and annotation characteristics.

#### 3.2.2. Contribution of Transfer Learning Under Different Sample Sizes

**Figure 6:**
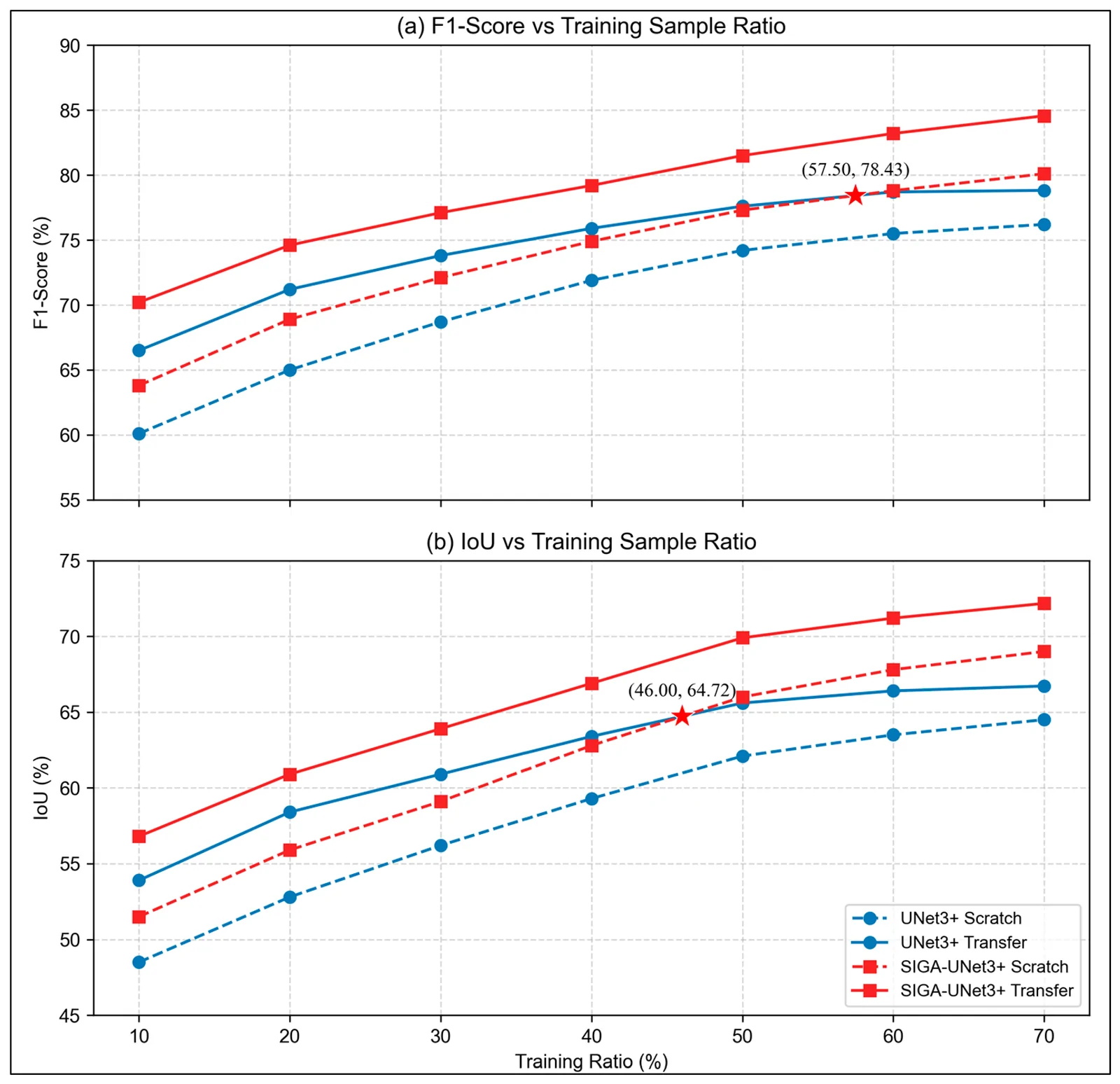
Transfer learning performance under different training sample ratios. The red star marks the intersection of the UNet3+ Transfer and SIGA-UNet3+ Scratch curves. The 10%, 20%, 30%, 40%, 50%, 60%, and 70% settings correspond to 276, 554, 830, 1107, 1384, 1661, and 1938 training patches, respectively. (**a**) F1-score results under different training sample ratios. (**b**) IoU results under different training sample ratios.

To further isolate the contribution of the transfer-learning strategy, we compared scratch training and transfer learning under identical network architectures at each training sample ratio. To evaluate the effectiveness of transfer learning under limited labeled-data conditions, the proportion of the complete GF-2 dataset used for training was varied from 10% to 70%. Given a total of 2768 image patches, the 10%, 20%, 30%, 40%, 50%, 60%, and 70% settings correspond to approximately 276, 554, 830, 1107, 1384, 1661, and 1938 training patches, respectively. The 70% setting corresponds to the complete training subset used in the comparative experiments in Section 3.2.1, while the validation and test subsets were kept unchanged. Figure 6a,b show the changing trends of F1-score and IoU under different experimental conditions.

As the proportion of training samples gradually increased, the curves of all four groups exhibited a continuous upward trend, indicating that increasing the amount of labeled data consistently improves building extraction performance. For example, the F1-score of UNet3+ trained from scratch increased from 60.17% to 76.20%, demonstrating that additional target-domain samples can effectively enhance the segmentation capability of the baseline model.

The paired scratch vs. transfer comparisons under the same network architecture provide a direct ablation of the transfer-learning strategy. For both UNet3+ and SIGA-UNet3+, transfer learning consistently improves F1-score and IoU across the evaluated sample ratios. When only 10% of the GF-2 training samples are used, transfer learning increases the F1-score by more than 6 pp and IoU by more than 5 pp relative to training the same architecture from scratch. As the amount of target-domain training data increases, the magnitude of this gain gradually decreases, reaching approximately 2–3 pp at the 70% setting. These results indicate that the benefit originates from the transfer-learning strategy itself rather than from differences in network architecture, and that its contribution is particularly pronounced under severe target-domain data scarcity.

Meanwhile, SIGA-UNet3+ consistently outperforms UNet3+ under both training strategies and across all sample ratios, demonstrating the contribution of the proposed spectral-guided attention module. Notably, the performance curve of SIGA-UNet3+ trained from scratch intersects with that of transfer-learning-based UNet3+ at specific sample ratios. As shown in Figure 6, the crossover point occurs at approximately 57.50% training samples for F1-score (F1 = 78.43%) and 46.00% training samples for IoU (IoU = 64.72%). Beyond these points, SIGA-UNet3+ trained from scratch achieves higher performance than transfer-learning-based UNet3+, suggesting that the proposed architectural improvement can partially compensate for the benefit of pre-training when sufficient target-domain samples are available.

Overall, Figure 6 demonstrates that training sample size, transfer learning strategy, and architectural improvement jointly influence model performance. Transfer learning is particularly beneficial under limited labeled-data conditions, whereas the proposed SIGA module provides consistent performance gains and reduces the dependence on large-scale pre-training when more target-domain samples become available.

## 4. Discussion

### 4.1. The Effectiveness of SIGA Module

The ablation experiments (Table 2) show that every single spectral index improves F1 by 1.87–3.57 percentage points (pp) over UNet3+. Notably, the ablation experiments in Table 2 provide quantitative evidence for the complementary roles of the three spectral indices. DSBI primarily improves Recall, indicating that it helps recover building regions that may otherwise be missed, whereas NDVI and NDWI mainly contribute to Precision improvement by suppressing false positives associated with vegetation and water. This behavior is consistent with the design motivation of SIGA: DSBI provides a positive building-enhancement prior, while NDVI and NDWI act as exclusionary priors for major non-building classes. The full three-index SIGA configuration achieves an additional 1.97 pp improvement in F1 over the best single-index variant, confirming that the indices provide complementary rather than fully redundant information. This result is consistent with recent studies demonstrating that fusing multiple spectral indices can improve built-up area extraction. For example, Ismaeel and Satish Kumar intersected seven indices (including NDVI, NDBI, BAEI, NBAI, NBI, MBI, and BRBA) to achieve a built-up map with 92.5% accuracy (Kappa = 0.848), and their subsequent DNN model reached over 95% prediction accuracy on Landsat 5 imagery, yielding a final Overall Accuracy of 92% and Kappa of 0.85 [48]. In our work, adding NDVI or NDWI to DSBI yields only marginal further gains (0.81 pp and 0.16 pp in F1), indicating diminishing returns due to partial overlap in the errors they correct. These findings indicate that the SIGA module effectively leverages complementary spectral priors to improve building extraction, with each index playing a distinct and synergistic role. More importantly, these physically interpretable spectral priors provide additional constraints beyond domain-specific texture features, which may improve the transferability of learned building representations under cross-domain conditions. Unlike fixed spectral-index filtering approaches, SIGA does not directly use spectral indices as hard constraints. Instead, the indices are incorporated as auxiliary priors to generate learnable attention weights, enabling adaptive feature recalibration through end-to-end optimization. However, the current ablation experiments mainly verify the contribution of spectral priors within SIGA and do not provide a direct controlled comparison with standard attention modules such as SE or CBAM.

Although SIGA-UNet3+ does not always achieve the highest Precision compared with transformer-based models, the results indicate that the proposed module improves the balance between false positives and false negatives. Specifically, the incorporation of spectral indices, particularly DSBI, enhances Recall by recovering building regions that are easily missed due to spectral ambiguity or boundary uncertainty. Meanwhile, NDVI and NDWI provide complementary information to suppress vegetation- and water-related false detections, thereby maintaining competitive Precision. This trade-off suggests that SIGA improves the balance between missed buildings and false detections rather than optimizing a single metric alone. By introducing material-related spectral information through spectral indices, SIGA provides additional guidance for feature recalibration and reduces the dependence on scene-specific spatial patterns, which is beneficial for building extraction across different regions and sensors. For building footprint extraction tasks, where missing building regions can directly affect subsequent geometric analysis, achieving a favorable Precision–Recall balance is more meaningful than maximizing a single metric.

The current combination of DSBI, NDVI, and NDWI demonstrates promising performance on the tested datasets. Still, there is room for further broadening: The current combination of DSBI, NDVI, and NDWI requires both visible and near-infrared bands because NDVI and NDWI depend on NIR information, which limits direct application of the complete SIGA configuration to RGB-only sensors. Future work will design a SWIR-free building index and introduce shadow-aware normalization, making the module adaptable to more sensor types and challenging conditions.

### 4.2. The Promoting Effect of Transfer Learning

The scratch vs. transfer comparisons in Figure 6 are performed using the same network architecture at each sample ratio, thereby separating the effect of the transfer-learning strategy from architectural differences.

The SpaceNet pre-trained model substantially improves building extraction under small-sample conditions: with only 10% of GF-2 training samples, transfer learning boosts F1 by 6–8 pp over training from scratch. This aligns with recent studies: Sawa et al. fine-tuned a Mask R-CNN pre-trained on US imagery using only 5–53 building instances in Ankara, achieving F1 scores of 0.92–0.96 across three SkySat satellites [49]; Neupane et al. proposed a fine-tuning-based transfer learning method for off-nadir images, reporting F1 scores of 0.868–0.943 for different building types [50]. Our experiments further reveal a non-linear saturation: the transfer learning gain drops from >6% at 10% samples to only 2–3 percentage points at 70% samples, indicating that pre-training is most valuable under extreme data scarcity (<20% of a typical dataset). Notably, SIGA-UNet3+ trained from scratch with 70% of GF-2 data surpasses UNet3+ with transfer learning at the same sample size, suggesting that the SIGA module’s architectural improvement can substitute for transfer learning when sufficient target data are available. Overall, transfer learning is particularly beneficial under severe data scarcity, while SIGA provides complementary architectural gains. Future work will further investigate how their joint use can be optimized under extremely limited target-domain annotation settings [51].

### 4.3. Limitations of the Study

**Comparative evaluation.** Although the current experiments include representative CNN-based and transformer-based segmentation models and verify the effectiveness of SIGA relative to the UNet3+ backbone, direct comparisons with a broader range of recent building-extraction-specific architectures and standard attention modules remain limited. Future work will extend these comparisons under unified training and evaluation settings, including recent building extraction methods and representative attention mechanisms such as SE, CBAM, and simple spatial or gated attention.

**Sensitivity to training and input settings.** The present study adopts equal weights of 0.5 and 0.5 for BCE and Dice losses and uses a fixed input size of 256 × 256 pixels to maintain consistent experimental conditions. A systematic sensitivity analysis of different loss-weight combinations and input patch sizes has not yet been conducted. Future work will further investigate these settings, including adaptive loss weighting and multiple input dimensions, to assess their influence on segmentation accuracy, contextual representation, and computational efficiency.

**Data and sensor generalization.** The experiments are based on two urban/suburban datasets (SpaceNet and GF-2). Therefore, the current experiments demonstrate cross-domain adaptability between the evaluated SpaceNet and GF-2 domains, rather than comprehensive generalization across all geographic regions, sensors, and scene types. To further evaluate the generalizability of SIGA-UNet3+ across more diverse scenarios, future work will include validation on more diverse scenes (e.g., rural, slum, or post-disaster areas, using datasets such as xBD and WHU). Moreover, the DSBI index is currently designed for sensors containing visible bands (blue, green, and red). Extending this formulation to incorporate additional spectral information, such as NIR or SWIR, will be explored in future work.

## 5. Conclusions

This study addressed two practical challenges in high-resolution remote sensing building extraction: the scarcity of labeled training data for target domains, and the limited cross-sensor and cross-region adaptability of conventional deep learning models. To overcome these challenges, we proposed SIGA-UNet3+, which integrates three physically based spectral indices (DSBI, NDVI, NDWI) through a gated attention mechanism, combined with a transfer learning strategy.

Experimental results demonstrate that SIGA-UNet3+ achieves better overall performance than the compared baseline models. On the SpaceNet dataset, it achieves an F1-score of 83.21% and IoU of 70.19%, representing improvements of 5.54 pp in F1 and 5.04 pp in IoU over UNet3+, and 1.24 pp and 1.74 pp over Swin Transformer. On the GF-2 dataset (different sensor and geographic region), it attains F1 = 84.56%, IoU = 72.17%, OA = 86.63%, and Kappa = 73.06%, again outperforming all compared baselines. Transfer learning with only 10% of GF-2 training samples improves F1 by 6–8 percentage points over training from scratch, indicating its potential value under limited-data conditions.

The scientific contributions of this work are threefold. First, we revealed the functional specialization of each index: DSBI primarily reduces missed buildings (Recall +8.10 pp), while NDVI and NDWI suppress false positives from vegetation and water (precision +2.24 pp and +1.16 pp, respectively). This provides an interpretation of why the triple combination achieves better performance. Second, the lightweight SIGA module adds only 0.4 M additional parameters and maintains inference time comparable to UNet3+ (27.3 ms vs. 26.8 ms), making it practical for real-world applications. Third, we identified a non-linear saturation of transfer learning gains (dropping from >6 percentage points at 10% samples to 2–3 percentage points at 70% samples) and a substitutive interaction between spectral priors and pre-training—insights that have direct implications for data-efficient model deployment.

Future work will focus on extending comparative evaluations with recent building-extraction-specific architectures and representative attention mechanisms, systematically investigating the sensitivity to loss weighting and input patch size, validating the model on more diverse geographic and scene conditions, and further improving the spectral-prior design for broader sensor applicability.

## Figures and Tables

**Table 1:** Quantitative results of different models on SpaceNet dataset ^1^.

Model	Precision/%	Recall/%	F1/%	IoU/%	OA/%	Kappa/%
Unet3+	80.30 ± 2.1	75.21 ± 1.8	77.67 ± 1.5	65.15 ± 3.2	81.12 ± 1.9	61.21 ± 2.0
SegNet	77.10 ± 2.0	72.48 ± 2.2	74.72 ± 1.9	59.67 ± 2.5	77.23 ± 2.1	53.86 ± 2.3
DeepLabv3+	79.47 ± 1.9	73.98 ± 1.7	76.63 ± 1.6	63.35 ± 2.1	80.05 ± 1.7	59.45 ± 1.8
Swin Transformer	85.12 ± 1.4	78.96 ± 1.5	81.97 ± 1.2	68.45 ± 2.3	84.02 ± 1.5	68.35 ± 1.7
SIGA-UNet3+	84.73 ± 1.3	81.74 ± 1.1	83.21 ± 1.0	70.19 ± 1.8	85.46 ± 1.2	71.36 ± 1.4

^1^ All results are reported as mean ± standard deviation (SD) based on three independent runs. The corresponding confidence intervals (CI) and effect sizes are provided in Appendix A (Table A1) to further evaluate performance variability and practical differences among models.

**Table 2:** Quantitative results of the ablation models.

Model	Precision/%	Recall/%	F1/%	IoU/%	OA/%	Kappa/%
Unet3+	80.30 ± 2.1	75.21 ± 1.8	77.67 ± 1.5	65.15 ± 3.2	81.12 ± 1.9	61.21 ± 2.0
DSBI-UNet3+	**79.28** ± 2.0	**83.31** ± 1.7	**81.24** ± 1.6	**68.86** ± 2.2	**83.58** ± 1.8	**67.08** ± 1.9
NDVI-UNet3+	82.54 ± 1.9	78.67 ± 1.6	80.56 ± 1.5	67.25 ± 2.0	82.96 ± 1.6	65.74 ± 1.7
NDWI-UNet3+	81.46 ± 2.0	77.72 ± 1.5	79.54 ± 1.4	65.97 ± 2.2	81.88 ± 1.7	63.49 ± 1.8
DSBI + NDVI	**82.15** ± 1.7	**82.07** ± 1.5	**82.05** ± 1.3	**69.53** ± 2.0	**84.38** ± 1.5	**68.19** ± 1.6
DSBI + NDWI	81.65 ± 1.8	81.21 ± 1.6	81.40 ± 1.3	68.94 ± 2.1	83.92 ± 1.6	66.94 ± 1.5
NDVI + NDWI	83.03 ± 1.6	80.52 ± 1.4	81.74 ± 1.2	69.28 ± 1.9	84.12 ± 1.4	68.22 ± 1.5
SIGA-UNet3+	**84.73** ± 1.3	**81.74** ± 1.1	**83.21** ± 1.0	**70.19** ± 1.8	**85.46** ± 1.2	**71.36** ± 1.4

Bold values indicate the best performance for each evaluation metric among the compared models.

**Table 3:** Computational cost comparison on SpaceNet dataset.

Model	Params (M)	FLOPs (G)	Inference Time (ms/Image)
Unet3+	27.1	78.3	26.8
SegNet	29.5	132.5	38.2
DeepLabv3+	27.8	85.6	31.5
Swin Transformer	28.3	92.7	41.5
SIGA-UNet3+	27.5	79.1	27.3

**Table 4:** Quantitative results of different models on GF-2 dataset ^1^.

Model	Precision/%	Recall/%	F1/%	IoU/%	OA/%	Kappa/%
Unet3+	81.42 ± 2.3	76.38 ± 1.9	78.82 ± 2.1	66.72 ± 3.4	82.17 ± 2.0	63.64 ± 2.2
SegNet	78.05 ± 2.4	73.41 ± 2.3	75.66 ± 2.2	60.98 ± 2.8	78.39 ± 1.9	56.01 ± 2.1
DeepLabv3+	80.63 ± 2.0	75.02 ± 1.8	77.72 ± 1.9	64.36 ± 2.5	80.94 ± 1.8	61.23 ± 1.9
Swin Transformer	87.45 ± 1.6	80.23 ± 1.4	83.53 ± 1.3	70.12 ± 2.1	85.16 ± 1.5	69.83 ± 1.6
SIGA-UNet3+	86.15 ± 1.5	83.02 ± 1.2	84.56 ± 1.1	72.17 ± 1.9	86.63 ± 1.3	73.06 ± 1.4

^1^ All results are reported as mean ± standard deviation (SD) based on three independent runs. The corresponding confidence intervals (CI) and effect sizes are provided in Appendix A (Table A2) to further evaluate performance variability and practical differences among models.

**Table 5:** BIoU comparison of different models on GF-2 dataset ^1^.

Model	BIoU/%
Unet3+	55.29 ± 3.5
SegNet	49.40 ± 3.1
DeepLabv3+	53.61 ± 2.8
Swin Transformer	61.27 ± 2.3
SIGA-UNet3+	64.79 ± 2.1

^1^ All results are reported as mean ± standard deviation (SD) based on three independent runs.

## Data Availability

The datasets used in this study are publicly available from their respective official platforms. The SpaceNet dataset is available through the official SpaceNet platform at https://spacenet.ai/datasets/ (accessed on 17 March 2025). The GF-2 imagery can be obtained from the China Centre for Resources Satellite Data and Application (CRESDA) at https://data.cresda.cn/ (accessed on 11 March 2025).

## Abbreviations

The following abbreviations are used in this manuscript:

SIGA	Spectral Index Gated Attention
SIGA-UNet3+	Spectral Index Gated Attention UNet3+
DSBI	Difference-based Spectral Built-up Index
NDVI	Normalized Difference Vegetation Index
NDWI	Normalized Difference Water Index
BCE	Binary Cross-Entropy
IoU	Intersection over Union
OA	Overall Accuracy

## Appendix A

This appendix presents two supplementary tables, each reporting the paired *t*-test *p*-values for Overall Accuracy (OA) and Kappa coefficient. All experiments were repeated three independent times, and the paired *t*-test (two-tailed, df = 2) was applied to compare the proposed SIGA-UNet3+ against baseline models.

**Table A1:** Confidence intervals and effect sizes of different models on the SpaceNet dataset.

Model	Metric	Mean ± SD (%)	95% CI (%) ^1^	Cohen’s d ^2^ (vs. SIGA-UNet3+)
UNet3+	F1	77.67 ± 1.5	[73.94, 81.40]	−4.35
DeepLabv3+	F1	76.63 ± 1.6	[72.66, 80.60]	−4.93
Swin Transformer	F1	81.97 ± 1.2	[78.99, 84.95]	−1.12
SIGA-UNet3+	F1	83.21 ± 1.0	[80.73, 85.69]	—
UNet3+	IoU	65.15 ± 3.2	[57.20, 73.10]	−1.94
DeepLabv3+	IoU	63.35 ± 2.1	[58.13, 68.57]	−3.50
Swin Transformer	IoU	68.45 ± 2.3	[62.74, 74.16]	−0.84
SIGA-UNet3+	IoU	70.19 ± 1.8	[65.72, 74.66]	—

^1^ The 95% confidence intervals (95% CI) were calculated based on three independent runs using the t-distribution (df = 2). ^2^ Cohen’s d represents the standardized mean difference between each compared model and SIGA-UNet3+, where negative values indicate that the compared model achieves lower performance than SIGA-UNet3+.

**Table A2:** Confidence intervals and effect sizes of different models on the GF-2 dataset.

Model	Metric	Mean ± SD (%)	95% CI (%) ^1^	Cohen’s d ^2^ (vs. SIGA-UNet3+)
UNet3+	F1	78.82 ± 2.1	[73.60, 84.03]	−3.43
DeepLabv3+	F1	77.72 ± 1.9	[73.00, 82224]	−4.41
Swin Transformer	F1	83.53 ± 1.3	[80.30, 86.76]	−0.86
SIGA-UNet3+	F1	84.56 ± 1.1	[81.83, 87.29]	—
UNet3+	IoU	66.72 ± 3.4	[58.27, 75.17]	−1.98
DeepLabv3+	IoU	64.36 ± 2.5	[58.15, 70.57]	−3.52
Swin Transformer	IoU	70.12 ± 2.1	[64.90, 75.34]	−0.98
SIGA-UNet3+	IoU	72.17 ± 1.9	[67.45, 76.89]	—

^1^ The 95% confidence intervals (95% CI) were calculated based on three independent runs using the t-distribution (df = 2). ^2^ Cohen’s d represents the standardized mean difference between each compared model and SIGA-UNet3+, where negative values indicate that the compared model achieves lower performance than SIGA-UNet3+.
